# HIV epidemic in Bulgaria

**DOI:** 10.1186/1742-4690-9-S1-P89

**Published:** 2012-05-25

**Authors:** Simon Shamas

**Affiliations:** 1 Merical University of Sofia, Sofia, Bulgaria

## Background

To identify the groups of highest risk of infection and understand the HIV dynamic in Bulgaria.

## Methods

By analyzing data presented by ''The National Center of Infectious and Parasitic Diseases'' about HIV/AIDS reported cases between 1986 and 2011.

## Results

During the period from 1986 to 2011 the number of registered patients is 1438 people. Last year the number of registered cases was 166, from which 81% are men. The annual number of officially registred HIV-infections increased from 49 in 1986 to 127 in 2007. We observe a development of HIV concentrated epidemics among the highest risk groups. Between 1986 and 2011 109 infections were due to Injecting drug use (IDU), the prevalence rate in this group was increased from 0.97% in 2004 to 7.29% in 2011. During the same period in the group of homosexuals the prevalence rate was increased from 0.99% to 1.6%. Romanies people are considered as a risk group because of their low social and economic status. Their prevalence rate reaches 2,9%. The newly diagnosed HIV-infections among prisoners in bulgarian jails increased 7,4 times between 2004 and 2007. In 2010 the lowest age limit of infection reached 15.

## Discussion

In Bulgaria HIV prevalence among the general population is below the national average for the countries of the EU. However, the country is facing a serious challenge in the form of rapid development of concentrated epidemics among some groups at higher risk. In order to protect the people there are some steps that should be taken as in motivating the groups of highest risk to use the services of voluntary free testing and counseling for HIV, rapid expansion of testing services through a network of Cabinets for anonymous and free counseling and assuring the access to knowledge about HIV/AIDS.

